# Differential expression and diagnostic significance of P53, MutS homologs 2, tropomyosin‐4 in alpha‐fetoprotein‐negative hepatocellular carcinoma

**DOI:** 10.1002/jcla.23353

**Published:** 2020-05-03

**Authors:** Xuyang Gong, Ailong Huang

**Affiliations:** ^1^ Infectious Disease and Molecular Biology Laboratory Chongqing Medical University Chongqing China

**Keywords:** alpha‐fetoprotein, hepatocellular carcinoma, MutS homologs 2, P53, tropomyosin‐4

## Abstract

**Background:**

Current study aimed to explore the value of P53, MutS homologs 2 (MSH2), and tropomyosin‐4 (Tm‐4) combined with inflammatory factors, life‐history traits in the differential diagnosis of alpha‐fetoprotein‐negative hepatocellular carcinoma (AFP‐Negative HCC).

**Methods:**

A testing cohort including 280 AFP‐Negative HCC patients and 300 controls was included. Three external validation cohorts from 3 centers were used to assess the novel logistic regression model including 400 AFP‐Negative HCC patients and 400 controls.

**Results:**

Compared with the control group, the levels of P53, MSH2, and Tm‐4 protein in si‐P53 group, si‐MSH2 group, and si‐Tm‐4 group were significantly reduced (*P* < .05). The P53, MSH2, Tm‐4, neutrophil to lymphocyte ratio (NLR), monocytes to lymphocyte ratio (MLR), hypersensitive C‐reactive protein (hs‐CRP), tumor necrosis factor‐α (TNF‐α), interleukin 6 (IL‐6) levels, and the smoking, drinking, and occupational exposure to chemicals rates in patients were significantly higher than those in controls (*P* < .05). ROC analyses showed that the area under curve (AUC) of NLR, MLR, hs‐CRP, TNF‐α, IL‐6, P53, MSH2, Tm‐4, drinking, smoking, and occupational exposure to chemicals were 0.798, 0.803, 0.560, 0.644, 0.808, 0.681, 0.830, 0.694, 0.582, 0.581, and 0.567, respectively. A novel logistic regression model was built and has a high value in identifying AFP‐Negative HCC with AUC of 0.917, sensitivity of 85.2%, and specificity of 88.3%. In the validation cohorts, this model also showed good diagnostic efficiency (AUC = 0.898 with Dazu Branch cohort, AUC = 0.924 with Jinshan Branch cohort, and AUC = 0.907 with Liangping Branch cohort).

**Conclusion:**

Current model has potential significance for the noninvasive diagnosis of AFP‐Negative HCC.

## 1 INTRODUCTION

Hepatocellular carcinoma (HCC) is the most common hepatic malignancy and has no obvious symptoms even in the middle stage.^1^, ^2^ Currently, the main mode of HCC screening is alpha‐fetoprotein (AFP).^3^ However, studies have shown that elevated serum AFP is observed in only 60% patients.^3^

Proteomics is an important part of post‐genomic research. With the help of proteomics research technology, the pathogenesis of tumors can be further revealed at the protein level, and tumor‐specific markers and specific antigens can be screened and identified. Recent studies have pointed out that abnormal expression of P53, MutS homologs 2 (MSH2), and tropomyosin‐4 (Tm‐4) was closely related to the occurrence and metastasis of various cancers.^4^, ^5^, ^6^, ^7^, ^8^ P53 is considered to be an important tumor suppressor protein, but reports have pointed out that the P53 gene in many cancer cells will mutate and lose cellular gene repair function.^4^ Wang et al^4^ pointed out that P53 gene was significantly increased in bladder cancer tissues, and high expression of P53 protein was associated with poor prognosis in patients. Recently, researchers found that the oncogene long non‐coding RNA H19 can play a competitive endogenous ribonucleic acid to inhibit miR‐29 expression, thereby reducing the inhibitory function of miR‐29 on P53 gene expression and promoting the cancer metastasis.^5^ MSH2 is an important type of mismatch repair protein. Hinrichsen et al^6^ cultured liver cancer HepG2 cell line in vitro and up‐regulated the expression of MSH2 by expressing MSH2 in HepG2 cells. They observed that up‐regulation of MSH2 can significantly up‐regulate the expression of the downstream cancer‐promoting target gene Krüppel‐like factor 4, thereby promoting the proliferation and migration of HepG2 cells and inhibiting apoptosis of cancer cells, suggesting that MSH2 can promote the biological activity of HepG2 cells. Differential expression of Tm‐4 participates in tumorigenesis and development by interfering with various biological behaviors.^7^, ^8^ Kabbage et al^7^ found that overexpression of Tm‐4 in breast cancer cell lines can inhibit tumor cell apoptosis through the p53‐mediated mitochondrial pathway and considered Tm‐4 as a new biomarker for breast cancer. However, the expression and diagnostic value of P53, MSH2, and Tm‐4 in patients with AFP‐Negative HCC have not been reported.

It is also well known that inflammation causes many cancers, especially liver cancer.^9^, ^10^ HCC is closely related to inflammation and usually occurs on the basis of chronic liver injury.^11^, ^12^ In this study, we developed a novel logistic regression model based on P53, MSH2, and Tm‐4 combined with inflammatory factors and life‐history traits to easily estimate AFP‐Negative HCC patients and validated this model in 3 external validation cohorts.

## 2 MATERIALS AND METHODS

### 2.1 Ethical approval

Informed patient consent was obtained in accordance with ethics committees of The First Affiliated Hospital of Chongqing Medical University (EA20150017).

### 2.2 Testing cohort

A total of 280 patients with AFP‐Negative HCC were consecutively selected at The First Affiliated Hospital of Chongqing Medical University from August 2016 and February 2019. Patients were diagnosed by puncture or intraoperative pathological section. We also consecutively included 300 subjects as the control group, including 160 patients with benign liver tumors (59 cases of liver hemangioma, 50 cases of liver cirrhosis nodules, and 51 cases of viral hepatitis) diagnosed at The First Affiliated Hospital of Chongqing Medical University and 140 healthy people from the Physical Examination Center of The First Affiliated Hospital of Chongqing Medical University.

The inclusion criteria were (a) the patient had not received any treatment such as radiotherapy and chemotherapy, (b) the patient diagnosed by histopathology, and (c) patient with good compliance. Exclusion criteria were (a) the patient who has not signed the informed consent, (b) the patient undergoing surgery, (c) the patient with other primary tumors, and (d) the patient with severe heart, liver, lung, and other organ dysfunction and patients with abnormal blood coagulation.

### 2.3 Validation cohorts

Three external validation cohorts from 3 centers (Dazu Branch, the First Affiliated Hospital of Chongqing Medical University; Jinshan Branch, the First Affiliated Hospital of Chongqing Medical University; and Liangping Branch, the First Affiliated Hospital of Chongqing Medical University) were used to assess the novel model including a total of 400 AFP‐Negative HCC patients and 400 controls. One hundred twenty‐five AFP‐Negative HCC and 130 controls, 140 AFP‐Negative HCC and 125 controls, and 135 AFP‐Negative HCC and 145 controls were recruited in these cohorts, respectively.

### 2.4 Biomarkers detection

Biomarkers tested in this study include inflammatory factors (neutrophil to lymphocyte ratio [NLR], platelet to lymphocyte ratio [PLR], monocytes to lymphocyte ratio [MLR], hypersensitive C‐reactive protein [hs‐CRP], tumor necrosis factor‐α [TNF‐α], and interleukin 6 [IL‐6]) and P53, MSH2, and Tm‐4 protein levels. NLR, PLR, and MLR are tested by BC‐5000 blood cell analyzer (Mindray). Hs‐CRP is tested by AU5400 (OLYMPUS). TNF‐α and IL‐6 are tested by E411 (Cobas). P53, MSH2, and Tm‐4 are tested by enzyme linked immunosorbent assay (ELISA; Thermo Fisher).

### 2.5 Cell culture and transfection

Hep G2 cells were cultured in Dulbecco's minimum essential medium (DMEM) containing 10% fetal bovine serum, 100 U/mL penicillin, and 100 μg/mL streptomycin and incubated in a 5% CO_2_ incubator at 37°C. Lipofectamine 2000 transfection reagent was used to transfect small interference RNA (si‐RNA) control, si‐P53, si‐MSH2, and si‐Tm‐4 into Hep G2 cells.

### 2.6 Western blot

The protein concentration of each sample was measured using the bicinchoninic acid (BCA) protein concentration measurement kit. The extracted protein was separated using sodium dodecyl sulfate‐polyacrylamide gel electrophoresis (SDS‐PAGE), and the protein of interest was transferred to a polyvinylidene fluoride (PVDF) membrane. Rabbit‐derived primary antibodies include P53 (code number: H00008030‐A01), MSH2 (code number: 3162‐100), Tm‐4 (code number: BS4007), and glyceraldehyde‐3‐phosphate dehydrogenase (GAPDH, code number: 10494‐1‐AP). A goat anti‐rabbit secondary antibody labeled with horseradish peroxidase (code number: A12004‐1) was used, and bands were measured using an enhanced chemiluminescence (ECL) kit.

### 2.7 Statistical analysis

SPSS 19.0 was performed. Data were presented as mean ± standard deviation (SD), or median [interquartile range (IQR)], or number of cases (%). Receiver operating characteristic (ROC) curve analysis was also used. The formula for calculating the number of diagnostic test samples is $n\;=\;{\left(\mu_{\alpha }^{2}/\delta \right)}^{2}\left(1-P\right)P$ where *α* is set to .05, *δ* is set to .05, and the *P* is set to 90%. *P* < .05 means the difference is statistically significant.

## 3 RESULTS

### 3.1 Inclusion of subjects and research process

**Figure 1:**
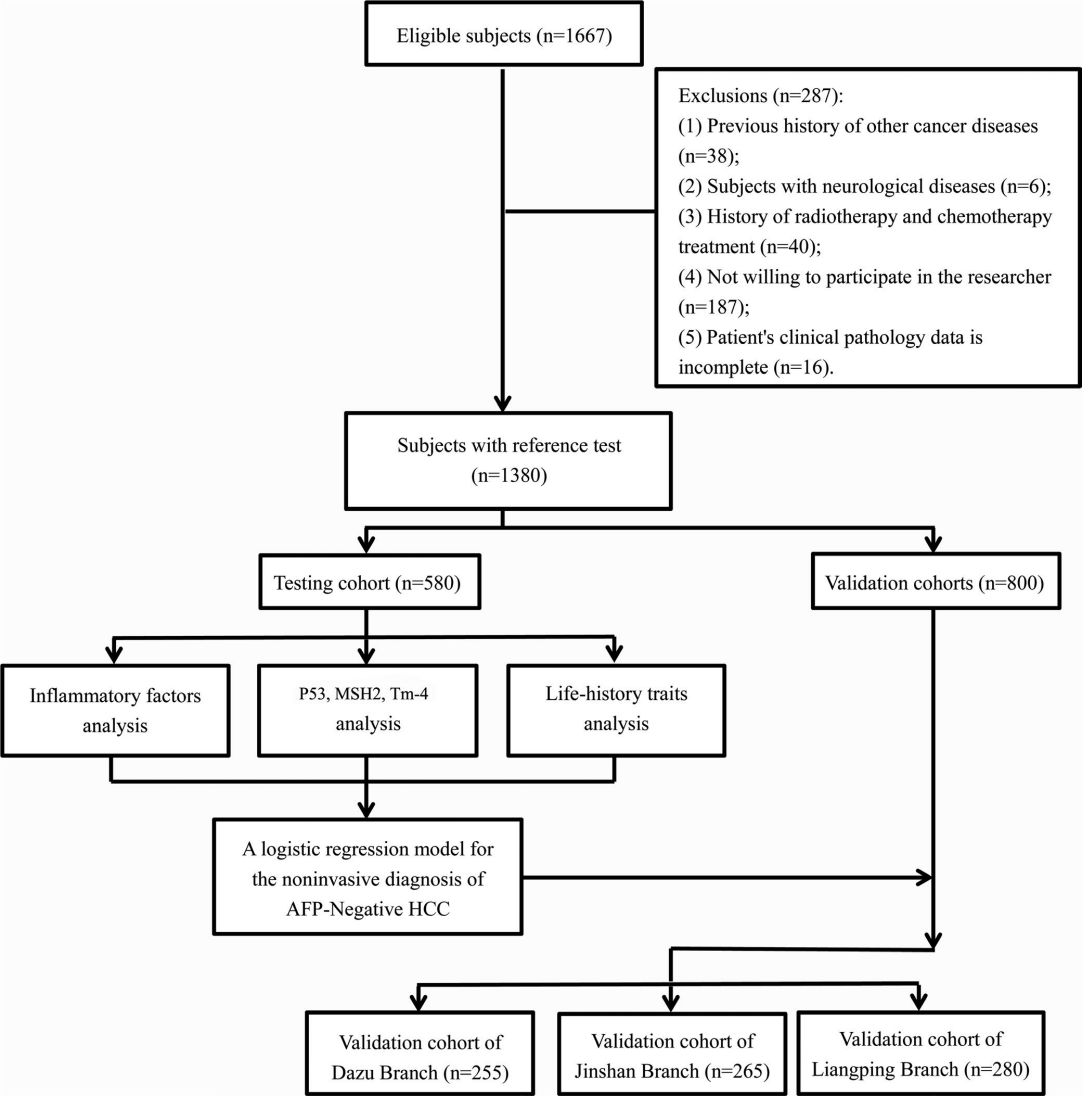
Flow diagram shows the inclusion and exclusion of eligible subjects

From August 2016 to February 2019, we identified 1667 eligible subjects at the First Affiliated Hospital of Chongqing Medical University. After exclusion of subjects who do not meet the inclusion criteria, 1380 subjects were included in the final analysis (Figure 1).

### 3.2 Specific verification of anti‐P53, anti‐MSH2, and anti‐Tm‐4 protein antibodies and levels of P53, MSH2, and Tm‐4 protein in AFP‐Negative HCC and adjacent tissues

**Figure 2:**
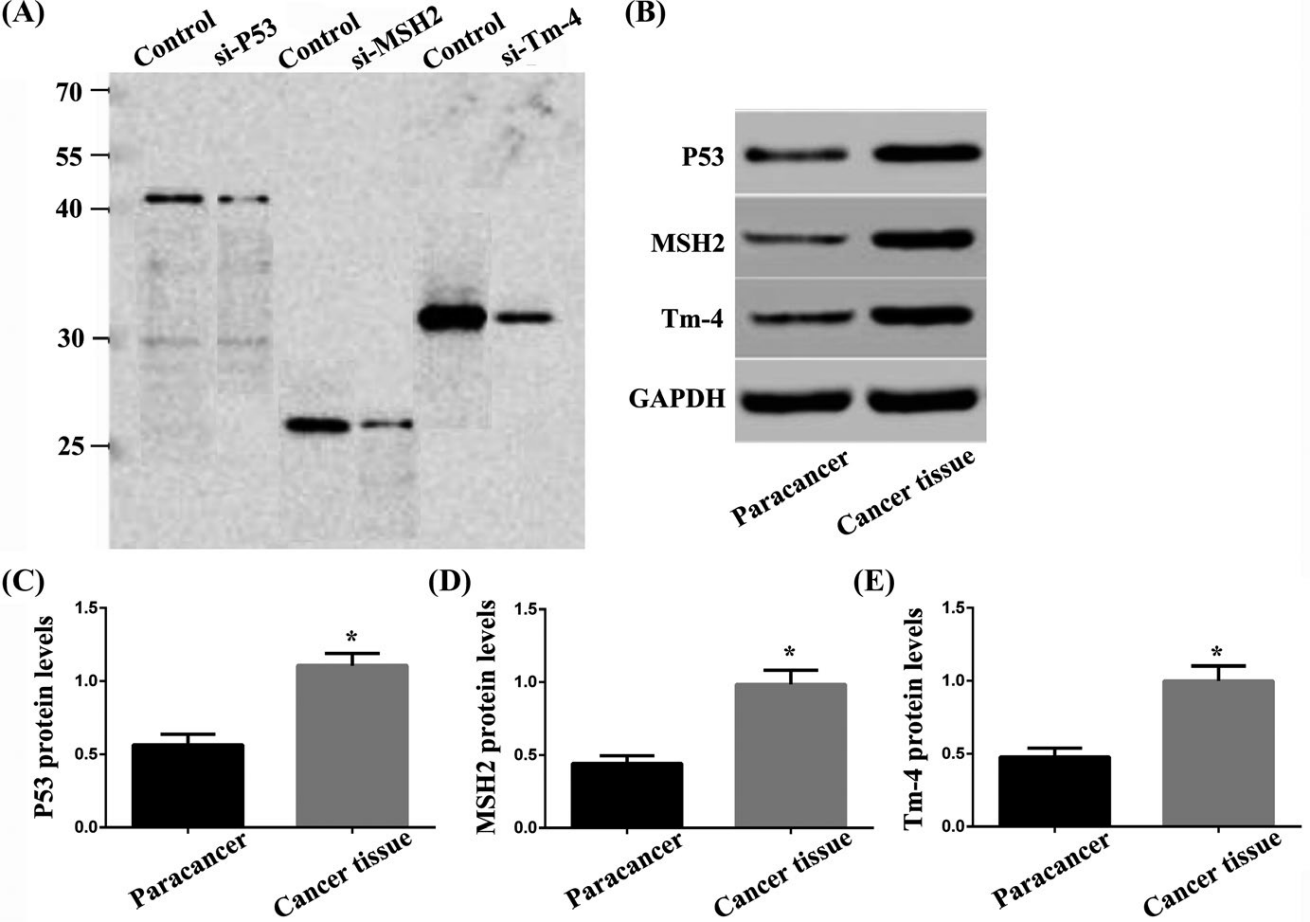
Specific verification of anti‐P53, anti‐MSH2, and anti‐Tm‐4 antibodies and P53, MSH2, and Tm‐4 protein levels in AFP‐Negative HCC and adjacent tissues. A, Compared with the control group, the levels of P53, MSH2, and Tm‐4 protein in si‐P53 group, si‐MSH2 group, and si‐Tm‐4 group were significantly reduced (*P* < .05). B‐E, P53, MSH2, and Tm‐4 protein levels in cancer tissues were significantly higher than those in adjacent tissues (*P* < .05)

The results of specific verification of anti‐P53, anti‐MSH2, and anti‐Tm‐4 protein antibodies are shown in Figure 2A. Compared with the control group, the levels of P53, MSH2, and Tm‐4 protein in si‐P53 group, si‐MSH2 group, and si‐Tm‐4 group were significantly reduced (*P* < .05). It is suggested that each antibody has high specificity, does not cross‐link with other proteins, and meets the experimental requirements. Western blot results showed that P53, MSH2, and Tm‐4 protein levels in cancer tissues were significantly higher than those in adjacent tissues (*P* < .05, Figure 2B‐E).

### 3.3 Expression of P53, MSH2, Tm‐4, inflammatory factors, and life‐history traits in testing cohort

**Figure 3:**
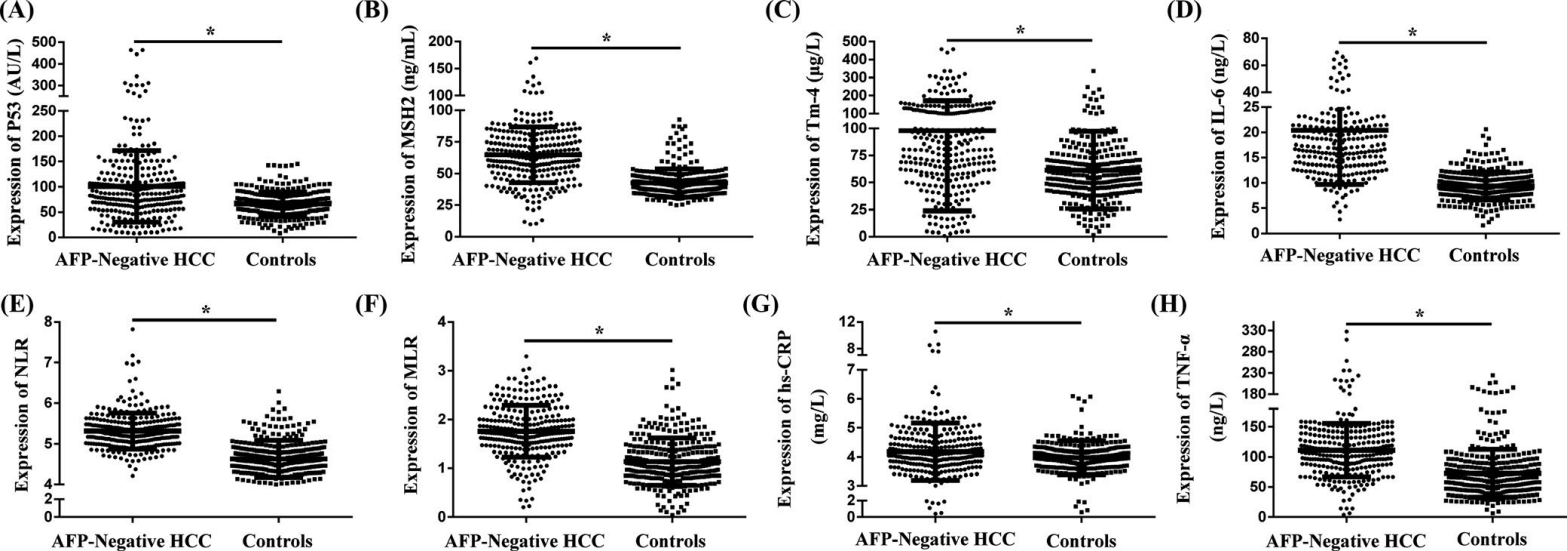
The P53, MutS homologs 2 (MSH2), and tropomyosin‐4 (Tm‐4), neutrophil to lymphocyte ratio (NLR), monocytes to lymphocyte ratio (MLR), hypersensitive C‐reactive protein (hs‐CRP), tumor necrosis factor‐α (TNF‐α), and interleukin 6 (IL‐6) levels in AFP‐Negative HCC patients, and controls. A, P53. B, MSH2. C, Tm‐4. D, IL‐6. E, NLR. F, MLR. G, hs‐CRP. H, TNF‐α. *P < .05

There were 580 subjects in the testing cohort. The subjects' P53, MSH2, and Tm‐4 protein levels, clinical information, inflammatory factors, and life‐history traits are presented in Table 1. The P53, MSH2, Tm‐4, NLR, MLR, hs‐CRP, TNF‐α, IL‐6 levels, and the smoking, drinking, and occupational exposure to chemicals rates in the AFP‐Negative HCC patients were significantly higher than those in controls (*P* < .05, Figure 3A‐H).

**Table 1:** P53, MSH2, and Tm‐4 protein levels, clinical information, inflammatory factors, and life‐history traits in the testing cohort

Variables	AFP‐Negative HCC patients	Controls	*χ* ^2^/*t*/*Z*	*P*
Number of subjects	280	300		
Clinical information				
Age, median (IQR)	53 (40, 68)	54 (42, 69)	1.746	.857
Male (%)	220 (78.6)	241 (80.3)	0.276	.600
Body mass index (mean ± SD, kg/m^2^)	22.9 ± 1.7	23.1 ± 1.9	−2.125	.183
Hypertension (%)	50 (17.9)	54 (18.0)	0.002	.964
Diabetes (%)	31 (11.1)	33 (11.0)	0.001	.978
Hyperlipidemia (%)	35 (12.5)	31 (10.3)	0.674	.412
Inflammatory factors				
NLR (mean ± SD)	5.3 ± 0.4	4.6 ± 0.5	4.723	<.001
PLR (mean ± SD)	153.3 ± 60.9	155.6 ± 63.5	−1.982	.657
MLR (mean ± SD)	1.7 ± 0.5	1.1 ± 0.5	5.003	<.001
hs‐CRP (mean ± SD, mg/L)	4.2 ± 1.0	3.8 ± 0.6	4.375	<.001
TNF‐α (mean ± SD, ng/L)	111.3 ± 44.2	92.1 ± 39.6	8.948	<.001
IL‐6 (mean ± SD, ng/L)	20.4 ± 8.9	9.4 ± 2.8	11.662	<.001
Protein markers				
P53 (mean ± SD, AU/L)	100.9 ± 64.4	65.8 ± 24.2	10.948	<.001
MSH2 (mean ± SD, ng/mL)	64.8 ± 22.1	42.5 ± 11.2	8.882	<.001
Tm‐4 (mean ± SD, ug/L)	98.2 ± 74.0	61.4 ± 36.0	9.150	<.001
Life‐history traits				
Drinking (%)	241 (86.1)	209 (69.7)	22.413	<.001
Smoking (%)	218 (77.9)	185 (61.7)	17.904	<.001
Family history of HCC (%)	26 (9.3)	21 (7.0)	1.106	.313
Occupational exposure to chemicals (%)	21 (7.5)	9 (3.0)	5.979	.014

Abbreviations: hs‐CRP, hypersensitive C‐reactive protein; IL‐6, interleukin 6; IQR, interquartile range; MLR, monocytes to lymphocyte ratio; MSH2, MutS homologs 2; NLR, neutrophil to lymphocyte ratio; PLR, platelet to lymphocyte ratio; SD, standard deviation; Tm‐4, tropomyosin‐4; TNF‐α, tumor necrosis factor‐α.

### 3.4 Logistic regression analyses and ROC analyses in testing cohort

Logistic regression analyses results are presented in Table 2. NLR (*P* < .001), MLR (*P* < .001), hs‐CRP (*P* = .037), TNF‐α (*P* = .002), IL‐6 (*P* = .010), P53 (*P* = .013), MSH2 (*P* < .001), Tm‐4 (*P* < .001) concentrations, drinking (*P* < .001), smoking (*P* < .001), and occupational exposure to chemicals (*P* < .001) had significant influence on AFP‐Negative HCC.

**Table 2:** Univariate and multivariate logistic regression analyses for AFP‐Negative HCC

Variables	Univariate analysis		Multivariate analysis	
	Odds ratio (95% CI)	*P*	Odds ratio (95% CI)	*P*
Age	1.056 (0.980, 1.149)	.608		
Male	0.944 (0.498, 1.772)	.824		
Body mass index	1.651 (0.925, 2.609)	.119		
Hypertension	1.094 (0.974, 1.242)	.226		
Diabetes	1.076 (0.516, 1.900)	.609		
Hyperlipidemia	0.991 (0.980, 1.084)	.617		
NLR	1.255 (1.097, 2.404)	<.001	1.264 (1.053, 2.902)	<.001
PLR	1.544 (0.763, 3.125)	.227		
MLR	1.937 (1.336, 2.896)	<.001	1.729 (1.111, 2.808)	<.001
hs‐CRP (mg/L)	1.021 (1.009, 1.034)	.002	1.006 (1.002, 1.015)	.037
TNF‐α (ng/L)	1.265 (1.060, 1.508)	<.001	1.093 (1.034, 1.156)	.002
IL‐6 (ng/L)	1.385 (1.177, 1.833)	<.001	1.099 (1.027, 1.168)	.010
P53 (AU/L)	1.112 (1.044, 2.516)	<.001	1.008 (1.003, 1.402)	.013
MSH2 (ng/mL)	1.075 (1.017, 1.995)	<.001	1.263 (1.096, 1.620)	<.001
Tm‐4 (ug/L)	1.817 (1.128, 2.926)	<.001	1.374 (1.054, 1.982)	<.001
Drinking	2.316 (1.562, 3.104)	<.001	2.024 (1.602, 2.437)	<.001
Smoking	1.804 (1.392, 2.337)	<.001	1.907 (1.425, 2.552)	<.001
Family history of HCC	1.265 (1.060, 1.508)	.009	1.003 (0.974, 1.156)	.142
Occupational exposure to chemicals	2.360 (1.555, 3.583)	<.001	1.709 (1.528, 2.761)	<.001

Abbreviations: hs‐CRP, hypersensitive C‐reactive protein; IL‐6, interleukin 6; MLR, monocytes to lymphocyte ratio; MSH2, MutS homologs 2; NLR, neutrophil to lymphocyte ratio; PLR, platelet to lymphocyte ratio; Tm‐4, tropomyosin‐4; TNF‐α, tumor necrosis factor‐α.

**Figure 4:**
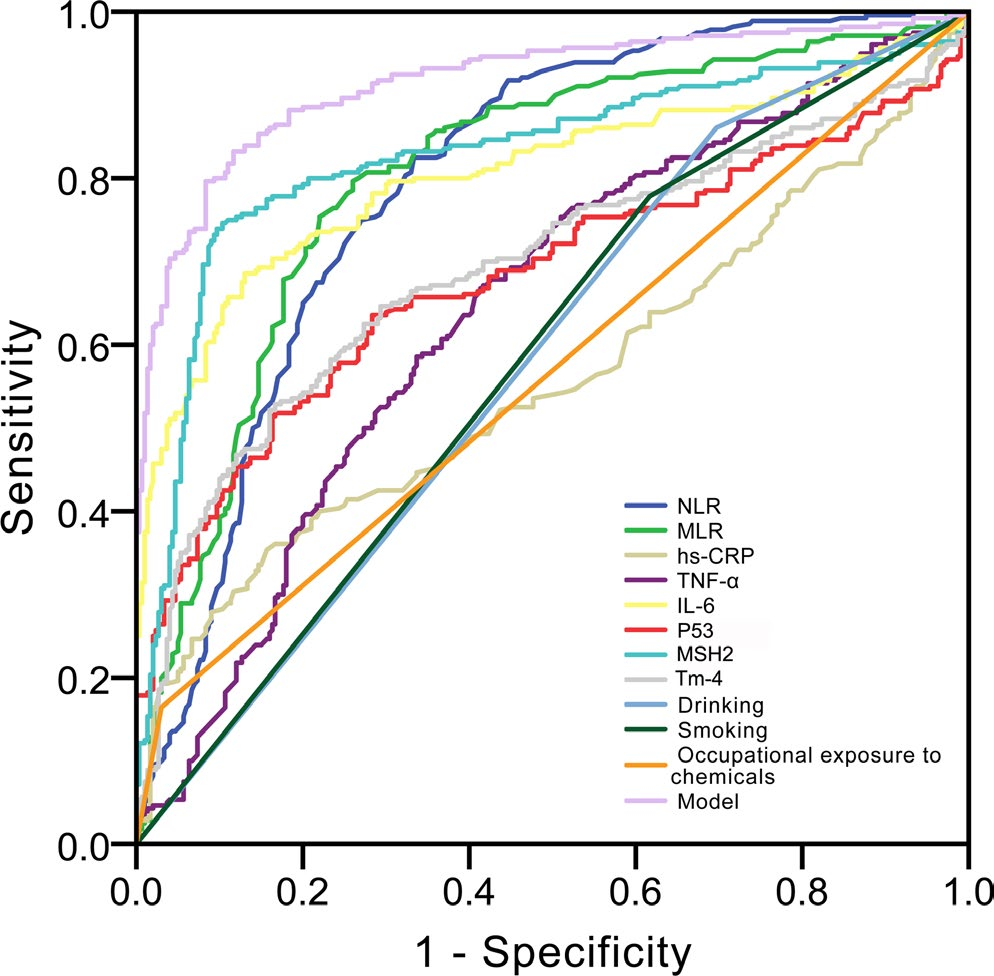
Receiver operating characteristic (ROC) curve analysis for the differential diagnosis values in the 11 independent factors [neutrophil to lymphocyte ratio (NLR), monocytes to lymphocyte ratio (MLR), hypersensitive C‐reactive protein (hs‐CRP), tumor necrosis factor‐α (TNF‐α), interleukin 6 (IL‐6), P53, MutS homologs 2 (MSH2), and tropomyosin‐4 (Tm‐4) concentrations, drinking, smoking, and occupational exposure to chemicals] for AFP‐Negative HCC

The area under curve (AUC) of NLR, MLR, hs‐CRP, TNF‐α, IL‐6, P53, MSH2, Tm‐4, drinking, smoking, and occupational exposure to chemicals were 0.798, 0.803, 0.560, 0.644, 0.808, 0.681, 0.830, 0.694, 0.582, 0.581, and 0.567, respectively (Figure 4 and Table S1).

### 3.5 A novel logistic regression model for AFP‐Negative HCC based on the testing cohort

The regression model for AFP‐Negative HCC was: Logit (*P*) = 707.379 − 7.167(NLR) − 23.925(MLR) − 17.162(hs‐CRP) − 0.041(TNF‐α) − 3.641(IL‐6) − 3.506(P53) − 0.679(MSH2) + 0.363(Tm‐4) − 99.383(drinking) − 172.883(smoking) − 179.393(occupational exposure to chemicals), see Table S2. The estimated probability was .316 (Figure 4 and Table S1).

### 3.6 Multicenter validation of the logistic regression model

The validity of our logistic regression model was assessed in 3 external validation cohorts. The subjects' data are presented in Table S3, Table S4, and Table S5. There were 800 subjects in the validation cohorts.

**Figure 5:**
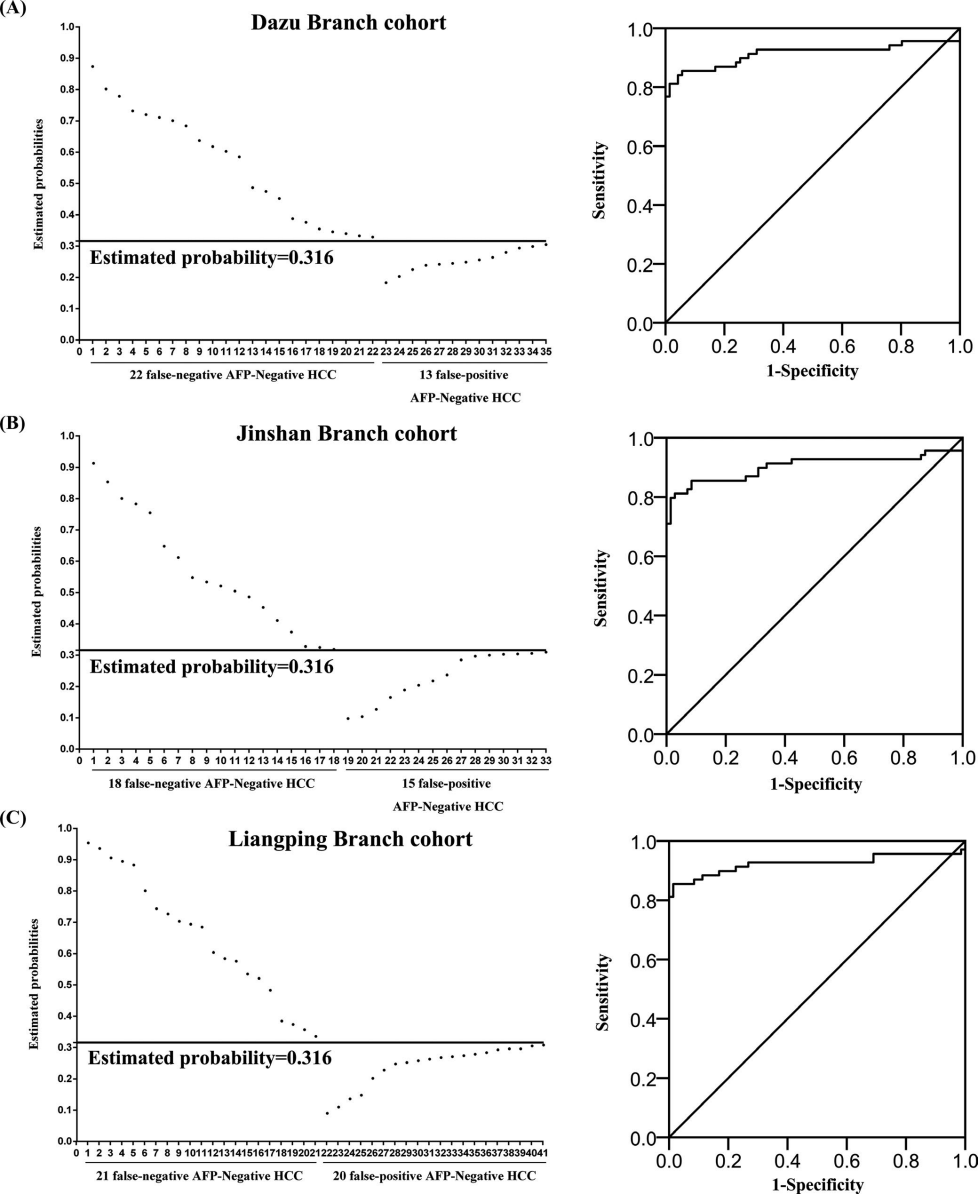
The scatter diagrams of the logistic regression model in 3 external validation cohorts. A, Twenty two false‐negative AFP‐negative patients with HCC and 13 false‐positive AFP‐negative patients with HCC in the Dazu Branch cohort. B, Eighteen false‐negative AFP‐negative patients with HCC and 15 false‐positive AFP‐negative patients with HCC in the Jinshan Branch cohort. C, Twenty one false‐negative AFP‐negative patients with HCC and 20 false‐positive AFP‐negative patients with HCC in the Liangping Branch cohort

In the Dazu Branch cohort, the sensitivity/specificity for AFP‐negative HCC was 82.4%/90.0%, with the AUC of 0.898 (95%CI: 0.865‐0.924), see Figure 5A. In the Jinshan Branch cohort, the sensitivity/specificity for AFP‐negative HCC was 87.1%/88.0%, with the AUC of 0.924 (95%CI: 0.897‐0.951), see Figure 5B. In the Liangping Branch cohort, the sensitivity/specificity for AFP‐negative HCC was 84.4%/86.2%, with the AUC of 0.907 (95%CI: 0.854‐0.916), see Figure 5C.

## 4 DISCUSSION

Hepatocellular carcinoma patients have rapid growth of tumor tissues, difficult to control the progression of the disease, and with poor prognosis. Because there is no effective targeted therapy, surgical treatment is still the main treatment for HCC. Although the clinical diagnosis and treatment technology of HCC has been significantly improved in recent years, the 5‐year survival rate of its patients has remained low.^13^ Studies have pointed out that molecular detection of proteomics technology has great application prospects for tumor prognosis evaluation. This study explores and establishes the diagnosis model for AFP‐negative HCC by combining P53, MSH2, and Tm‐4 protein molecules, with inflammation markers. Recent studies have pointed out that the abnormal expression of proteins such as P53, MSH2, and Tm‐4 was closely related to the occurrence and metastasis of HCC.^4^, ^5^, ^6^, ^7^, ^8^ Moreover, the inflammatory response is self‐limiting under normal conditions, as anti‐inflammatory cytokines are expressed rapidly after expression of pro‐inflammatory factors.^14^, ^15^, ^16^, ^17^, ^18^ In malignant tumors, especially liver cancer, the continuation of the inflammatory response is mainly caused by the continuous stimulation of chronic viral infection.^11^, ^19^, ^20^ The persistent microenvironment of inflammation often promotes tumor progression and plays an important role in the metastasis of tumors.

In this study, our results indicated that the NLR, MLR, hs‐CRP, TNF‐α, IL‐6, P53, MSH2, and Tm‐4 levels and the smoking, drinking, and occupational exposure to chemicals rates in the AFP‐Negative HCC patients were significantly higher than those in controls. Up‐regulation of peripheral neutrophils and monocytes are thought to reflect the intrinsically aggressive nature of tumor cells because they are induced by cytokines produced by tumor cells.^21^, ^22^, ^23^ P53 has high specificity for the diagnosis of primary liver cancer.^24^, ^25^ Previous studies have shown that MSH2 is closely related to the development of various liver diseases, especially HCC.^26^, ^27^ It has been reported in China that the expression of MSH2 is gradually up‐regulated during the development of liver disease. MSH2 is hardly expressed in normal liver cells, but may increase slightly in the presence of acute inflammation or more severe liver fibrosis in the liver, and the expression of MSH2 may rapidly increase and reach a high level when it develops into HCC.^28^ Therefore, the level of MSH2 is considered to be closely related to the process of HCC and may become a candidate marker for HCC. The increase in Tm‐4 mainly includes an increase in the source of ferritin or a clearing disorder. Among them, the increase of synthetic Tm‐4 in cancer cells is an important source.^29^ In our research, MSH2 was the most effective indicator (AUC = 0.830) for the diagnosis of AFP‐negative HCC than NLR (AUC = 0.798), MLR (AUC = 0.803), hs‐CRP (AUC = 0.560), TNF‐α (AUC = 0.644), IL‐6 (AUC = 0.808), P53 (AUC = 0.681), Tm‐4 (AUC = 0.694), drinking (AUC = 0.582), smoking (AUC = 0.581), and occupational exposure to chemicals (AUC = 0.567), but its sensitivity(70.9%) was unsatisfactory.

Therefore, we built a novel logistic regression model containing NLR, MLR, hs‐CRP, TNF‐α, IL‐6, P53, MSH2, Tm‐4, drinking, smoking, and occupational exposure to chemicals. It showed higher diagnostic efficiency (AUC = 0.917, sensitivity = 85.2%, specificity = 88.3%) than any single parameter. Of course, the model that combined different parameters has been reported.^30^ However, we found that current model has better AUC (0.917) than the model of combing AFP and DCP (AUC = 0.910).^30^ Furthermore, we validated this model in 3 validation cohorts. In order to avoid the occurrence of selection bias as much as possible, we selected patients and controls from three different branches. This model showed good diagnostic efficiency in all validation cohorts (AUC = 0.898 with Dazu Branch cohort, AUC = 0.924 with Jinshan Branch cohort, and AUC = 0.907 with Liangping Branch cohort), indicating that this model is able to predict AFP‐Negative HCC.

In summary, current model including P53, MSH2, Tm‐4, inflammatory factors, and life‐history traits might more effectively improve the diagnostic efficiency of AFP‐Negative HCC.

## AUTHOR CONTRIBUTIONS

AH researched literature and conceived the study. XG and AH were involved in gaining ethical approval, patient recruitment, and data analysis. XG wrote the first draft of the manuscript. All authors reviewed and edited the manuscript and approved the final version of the manuscript.

## ETHICAL APPROVAL

The ethics committee of The First Affiliated Hospital of Chongqing Medical University approved this study (EA20150017).

## Supporting information

Table S1‐S5
