# Supplementary material for: Differential expression and diagnostic significance of P53, MutS homologs 2, tropomyosin‐4 in alpha‐fetoprotein‐negative hepatocellular carcinoma
Source: J Clin Lab Anal. 2020 May 3;34(8):e23353. doi: 10.1002/jcla.23353 (PMC7439328; doi:10.1002/jcla.23353)
Supplement: Supplementary file 1 — Table S1‐S5 [file JCLA-34-e23353-s001.docx]

**Supplementary Table 1** The calculated performance values for different variables in the testing group (n=280).

| Variable | AUC | 95% *CI* | *P* | Sensitivity (%) | Specificity (%) |
| --- | --- | --- | --- | --- | --- |
| NLR | 0.798 | 0.762-0.834 | <0.001 | 82.5 | 66.7 |
| MLR | 0.803 | 0.767-0.840 | <0.001 | 79.6 | 74.0 |
| hs-CRP | 0.560 | 0.512-0.608 | 0.013 | 35.4 | 84.3 |
| TNF-α | 0.644 | 0.600-0.689 | <0.001 | 66.8 | 58.7 |
| IL-6 | 0.808 | 0.771-0.845 | <0.001 | 68.6 | 87.0 |
| P53 | 0.681 | 0.636-0.726 | <0.001 | 63.6 | 71.7 |
| MSH2 | 0.830 | 0.793-0.866 | <0.001 | 70.9 | 89.7 |
| Tm-4 | 0.694 | 0.649-0.738 | <0.001 | 52.9 | 83.3 |
| Drinking | 0.582 | 0.536-0.628 | 0.001 | 84.1 | 30.3 |
| Smoking | 0.581 | 0.535-0.627 | 0.001 | 77.9 | 38.3 |
| Occupational exposure to chemicals | 0.567 | 0.520-0.614 | 0.005 | 16.4 | 97.0 |
| Model | 0.917 | 0.893-0.941 | <0.001 | 85.2 | 88.3 |

Abbreviation: NLR: neutrophil to lymphocyte ratio; PLR: platelet to lymphocyte ratio; MLR: monocytes to lymphocyte ratio; hs-CRP: hypersensitive C-reactive protein; TNF-α: tumor necrosis factor-α; IL-6: interleukin 6; MSH2: MutS homologs 2; Tm-4: tropomyosin-4.

**Supplementary Table 2** A logistic regression model for the AFP-Negative HCC prediction

| Variable | Influence coefficient (β) | Standard error | Wald |
| --- | --- | --- | --- |
| NLR | -7.167 | 4.572 | 2.458 |
| MLR | -23.925 | 19.629 | 1.486 |
| hs-CRP | -17.162 | 15.197 | 1.275 |
| TNF-α | -0.041 | 0.066 | 0.386 |
| IL-6 | -3.641 | 3.025 | 1.448 |
| P53 | -3.506 | 2.981 | 1.383 |
| MSH2 | -0.679 | 0.521 | 1.698 |
| Tm-4 | 0.363 | 0.311 | 1.359 |
| Drinking | -99.383 | 26134.62 | 0 |
| Smoking | -172.883 | 26135.07 | 0 |
| Occupational exposure to chemicals | -179.393 | 153.965 | 1.358 |
| Constant | 707.379 | 578.979 | 1.493 |

Abbreviation: NLR: neutrophil to lymphocyte ratio; PLR: platelet to lymphocyte ratio; MLR: monocytes to lymphocyte ratio; hs-CRP: hypersensitive C-reactive protein; TNF-α: tumor necrosis factor-α; IL-6: interleukin 6; MSH2: MutS homologs 2; Tm-4: tropomyosin-4.

**Supplementary Table 3** Clinical information, inflammatory factors, P53, MSH2, Tm-4, and life-history traits in the validation cohort (Dazu Branch)

| Variables |  | AFP-Negative HCC patients | Controls | *χ^2^/t/Z* | *P* |
| --- | --- | --- | --- | --- | --- |
|  | Number of subjects | 125 | 130 |  |  |
| Clinical information |  |  |  |  |  |
|  | Age, median (IQR) | 50 (41, 67) | 53 (44, 68) | 1.362 | 0.407 |
|  | Male (%) | 103 (82.4) | 106 (81.5) | 0.866 | 0.352 |
|  | Body mass index (mean ± SD, kg/m^2^) | 23.1±1.5 | 23.0±1.4 | 0.038 | 0.982 |
|  | Hypertension (%) | 27 (21.6) | 24 (18.5) | 0.392 | 0.531 |
|  | Diabetes (%) | 13 (10.4) | 16 (12.3) | 0.230 | 0.631 |
|  | Hyperlipidemia (%) | 10 (8.0) | 12 (9.2) | 0.122 | 0.726 |
| Inflammatory factors |  |  |  |  |  |
|  | NLR (mean ± SD) | 5.7±0.5 | 4.3±0.4 | 4.971 | <0.001 |
|  | PLR (mean ± SD) | 150.1±59.0 | 149.6±55.7 | 2.017 | 0.145 |
|  | MLR (mean ± SD) | 1.9±0.7 | 1.2±0.3 | 8.944 | <0.001 |
|  | hs-CRP (mean ± SD, mg/L) | 4.0±0.6 | 3.5±0.4 | 3.962 | <0.001 |
|  | TNF-α (mean ± SD, ng/L) | 117.4±40.3 | 90.5±37.0 | 6.664 | <0.001 |
|  | IL-6 (mean ± SD, ng/L) | 21.0±7.9 | 9.9±2.1 | 11.300 | <0.001 |
| Protein markers |  |  |  |  |  |
|  | P53 (mean ± SD, AU/L) | 94.3±40.7 | 63.2±20.5 | 10.021 | <0.001 |
|  | MSH2 (mean ± SD, ng/mL) | 70.9±20.4 | 49.0±14.8 | 9.917 | <0.001 |
|  | Tm-4 (mean ± SD, ug/L) | 112.2±58.0 | 69.0±30.2 | 13.927 | <0.001 |
| Life-history traits |  |  |  |  |  |
|  | Drinking (%) | 108 (86.4) | 88 (67.7) | 12.541 | <0.001 |
|  | Smoking (%) | 103 (82.4) | 78 (60.0) | 15.523 | <0.001 |
|  | Family history of HCC (%) | 12 (9.6) | 14 (10.8) | 0.095 | 0.758 |
|  | Occupational exposure to chemicals (%) | 11 (8.8) | 2 (1.5) | 6.998 | 0.008 |

Abbreviation: SD: standard deviation; IQR: interquartile range; NLR: neutrophil to lymphocyte ratio; PLR: platelet to lymphocyte ratio; MLR: monocytes to lymphocyte ratio; hs-CRP: hypersensitive C-reactive protein; TNF-α: tumor necrosis factor-α; IL-6: interleukin 6; MSH2: MutS homologs 2; Tm-4: tropomyosin-4.

**Supplementary Table 4** Clinical information, inflammatory factors, P53, MSH2, Tm-4, and life-history traits in the validation cohort (Jinshan Branch)

| Variables |  | AFP-Negative HCC patients | Controls | *χ^2^/t/Z* | *P* |
| --- | --- | --- | --- | --- | --- |
|  | Number of subjects | 140 | 125 |  |  |
| Clinical information |  |  |  |  |  |
|  | Age, median (IQR) | 49 (38, 69) | 50 (39, 67) | 0.781 | 0.774 |
|  | Male (%) | 105 (75.0) | 96 (76.8) | 0.117 | 0.733 |
|  | Body mass index (mean ± SD, kg/m^2^) | 22.8±1.6 | 22.7±1.2 | 0.105 | 0.569 |
|  | Hypertension (%) | 22 (15.7) | 20 (16.0) | 0.004 | 0.949 |
|  | Diabetes (%) | 14 (10.0) | 15 (12.0) | 0.271 | 0.603 |
|  | Hyperlipidemia (%) | 15 (10.7) | 12 (9.6) | 0.090 | 0.765 |
| Inflammatory factors |  |  |  |  |  |
|  | NLR (mean ± SD) | 5.0±0.5 | 4.4±0.3 | 5.246 | <0.001 |
|  | PLR (mean ± SD) | 148.0±39.1 | 148.6±33.0 | 0.099 | 0.893 |
|  | MLR (mean ± SD) | 2.0±0.5 | 1.2±0.4 | 5.322 | <0.001 |
|  | hs-CRP (mean ± SD, mg/L) | 4.7±1.3 | 3.6±0.9 | 5.983 | <0.001 |
|  | TNF-α (mean ± SD, ng/L) | 124.3±37.1 | 86.9±22.7 | 8.066 | <0.001 |
|  | IL-6 (mean ± SD, ng/L) | 19.0±5.5 | 10.9±3.2 | 12.741 | <0.001 |
| Protein markers |  |  |  |  |  |
|  | P53 (mean ± SD, AU/L) | 92.7±28.0 | 62.5±20.2 | 13.002 | <0.001 |
|  | MSH2 (mean ± SD, ng/mL) | 61.9±17.0 | 40.4±12.8 | 9.413 | <0.001 |
|  | Tm-4 (mean ± SD, ug/L) | 86.0±41.8 | 67.0±33.9 | 7.075 | <0.001 |
| Life-history traits |  |  |  |  |  |
|  | Drinking (%) | 126 (90.0) | 92 (73.6) | 12.174 | <0.001 |
|  | Smoking (%) | 113 (80.7) | 74 (59.2) | 14.716 | <0.001 |
|  | Family history of HCC (%) | 8 (5.7) | 7 (5.6) | 0.002 | 0.968 |
|  | Occupational exposure to chemicals (%) | 13 (9.3) | 3 (2.4) | 5.519 | 0.019 |

Abbreviation: SD: standard deviation; IQR: interquartile range; NLR: neutrophil to lymphocyte ratio; PLR: platelet to lymphocyte ratio; MLR: monocytes to lymphocyte ratio; hs-CRP: hypersensitive C-reactive protein; TNF-α: tumor necrosis factor-α; IL-6: interleukin 6; MSH2: MutS homologs 2; Tm-4: tropomyosin-4.

**Supplementary Table 5** Clinical information, inflammatory factors, P53, MSH2, Tm-4, and life-history traits in the validation cohort (Liangping Branch)

| Variables |  | AFP-Negative HCC patients | Controls | *χ^2^/t/Z* | *P* |
| --- | --- | --- | --- | --- | --- |
|  | Number of subjects | 135 | 145 |  |  |
| Clinical information |  |  |  |  |  |
|  | Age, median (IQR) | 54 (45, 69) | 52 (44, 72) | 1.305 | 0.197 |
|  | Male (%) | 110(81.5) | 121 (83.4) | 0.187 | 0.665 |
|  | Body mass index (mean ± SD, kg/m^2^) | 23.4±1.2 | 23.3±1.5 | 0.223 | 0.540 |
|  | Hypertension (%) | 26 (19.3) | 25 (17.2) | 0.191 | 0.662 |
|  | Diabetes (%) | 15 (11.1) | 19 (13.1) | 0.260 | 0.610 |
|  | Hyperlipidemia (%) | 19 (14.1) | 17 (11.7) | 0.345 | 0.557 |
| Inflammatory factors |  |  |  |  |  |
|  | NLR (mean ± SD) | 5.2±0.3 | 4.4±0.4 | 6.107 | <0.001 |
|  | PLR (mean ± SD) | 148.7±43.2 | 147.9±40.6 | 0.126 | 0.873 |
|  | MLR (mean ± SD) | 1.9±0.4 | 1.3±0.2 | 6.663 | <0.001 |
|  | hs-CRP (mean ± SD, mg/L) | 4.7±1.1 | 4.0±0.8 | 4.028 | <0.001 |
|  | TNF-α (mean ± SD, ng/L) | 104.7±38.4 | 94.4±28.9 | 3.979 | 0.011 |
|  | IL-6 (mean ± SD, ng/L) | 19.7±6.7 | 10.4±4.9 | 8.550 | <0.001 |
| Protein markers |  |  |  |  |  |
|  | P53 (mean ± SD, AU/L) | 95.4±28.6 | 71.4±20.0 | 7.904 | <0.001 |
|  | MSH2 (mean ± SD, ng/mL) | 65.7±17.1 | 46.8±12.8 | 6.936 | <0.001 |
|  | Tm-4 (mean ± SD, ug/L) | 105.6±53.8 | 73.9±49.2 | 9.480 | <0.001 |
| Life-history traits |  |  |  |  |  |
|  | Drinking (%) | 110 (81.5) | 91 (62.8) | 12.100 | <0.001 |
|  | Smoking (%) | 98 (72.6) | 85 (58.6) | 6.028 | 0.014 |
|  | Family history of HCC (%) | 7 (5.2) | 11 (7.6) | 0.670 | 0.413 |
|  | Occupational exposure to chemicals (%) | 15 (11.1) | 6 (4.1) | 4.900 | 0.027 |

Abbreviation: SD: standard deviation; IQR: interquartile range; NLR: neutrophil to lymphocyte ratio; PLR: platelet to lymphocyte ratio; MLR: monocytes to lymphocyte ratio; hs-CRP: hypersensitive C-reactive protein; TNF-α: tumor necrosis factor-α; IL-6: interleukin 6; MSH2: MutS homologs 2; Tm-4: tropomyosin-4.
